# Increased Methylglyoxal Formation with Upregulation of Renin Angiotensin System in Fructose Fed Sprague Dawley Rats

**DOI:** 10.1371/journal.pone.0074212

**Published:** 2013-09-10

**Authors:** Indu Dhar, Arti Dhar, Lingyun Wu, Kaushik M. Desai

**Affiliations:** 1 Department of Pharmacology, College of Medicine, University of Saskatchewan, Saskatoon, Saskatchewan, Canada; 2 Department of Health Sciences, Lakehead University, Thunder Bay, Ontario, Canada; 3 Thunder Bay Regional Research Institute, Thunder Bay, Ontario, Canada; The University of Manchester, United Kingdom

## Abstract

The current epidemic of obesity and type 2 diabetes is attributed to a high carbohydrate diet, containing mainly high fructose corn syrup and sucrose. More than two thirds of diabetic patients have hypertension. Methylglyoxal is a highly reactive dicarbonyl generated during glucose and fructose metabolism, and a major precursor of advanced glycation end products (AGEs). Plasma methylglyoxal levels are increased in hypertensive rats and diabetic patients. Our aim was to examine the levels of methylglyoxal, mediators of the renin angiotensin system and blood pressure in male Sprague-Dawley rats treated with a high fructose diet (60% of total calories) for 4 months. The thoracic aorta and kidney were used for molecular studies, along with cultured vascular smooth muscle cells (VSMCs). HPLC, Western blotting and Q-PCR were used to measure methylglyoxal and reduced glutathione (GSH), proteins and mRNA, respectively. Fructose treated rats developed a significant increase in blood pressure. Methylglyoxal level and protein and mRNA for angiotensin II, AT_1_ receptor, adrenergic α_1D_ receptor and renin were significantly increased, whereas GSH levels were decreased, in the aorta and/or kidney of fructose fed rats. The protein expression of the receptor for AGEs (RAGE) and NF-κB were also significantly increased in the aorta of fructose fed rats. MG treated VSMCs showed increased protein for angiotensin II, AT_1_ receptor, and α_1D_ receptor. The effects of methylglyoxal were attenuated by metformin, a methylglyoxal scavenger and AGEs inhibitor. In conclusion, we report a strong association between elevated levels of methylglyoxal, RAGE, NF-κB, mediators of the renin angiotensin system and blood pressure in high fructose diet fed rats.

## Introduction

High dietary carbohydrates, increasing type 2 diabetes and obesity, and the associated hypertension and cardiovascular diseases are major health issues globally [1], [2], [3]. The explosive increase in type 2 diabetes in the past 2–3 decades has been attributed to high dietary carbohydrates, especially fructose, combined with a sedentary lifestyle. Compared to the general population the risk of high blood pressure (BP, systolic BP≥140 mmHg or diastolic BP≥90 mmHg) is four times higher for people with diabetes [4]. The pathogenesis of diabetes-associated hypertension is not clearly known. While it is believed to be initiated by hyperglycemia, the molecular mechanisms are far from clear.

The Western diet has high fructose content, mainly in the form of high fructose corn syrup, which has been proposed to induce hypertension [1], [3]. High fructose diet-fed Sprague-Dawley (SD) rats have been widely used as a model of insulin resistance and these rats also develop hypertension [5], [6].

We have shown that fructose-fed SD rats have elevated levels of methylglyoxal (MG), a reactive metabolite of glucose and fructose [6], [7]. Elevated plasma levels of MG have been reported in spontaneously hypertensive rats which correlate with the degree of hypertension [8], [9], but the cause-effect relationship and the underlying molecular mechanisms are not known. People with diabetes have significantly elevated levels of MG [10], [11]. We have recently reported that chronic MG induces features of type 2 diabetes in SD rats [12]. MG is a major precursor for the formation of advanced glycation end products (AGEs) [13], [14]. MG reduces activity of antioxidant enzymes like glutathione reductase and glutathione peroxidase [15], leading to increased oxidative stress, which in turn is believed to cause the pathophysiological changes in diabetes, hypertension, and aging [16], [17]. The pathogenesis of hypertension is multifactorial. Some of the factors include an increase in renin angiotensin aldosterone system (RAAS) activity, insulin resistance, renal disease and oxidative stress [18], [19], [20], [21]. The RAAS plays an important role in maintaining fluid balance, vascular tone and blood pressure [22], [23]. MG and angiotensin II (Ang II) both lead to an increase in oxidative stress [21], [24], [25]. Ang II stimulates NADPH oxidase by acting through the AT_1_ receptor and increases superoxide, hydrogen peroxide, and peroxynitrite [24]. However, the cause and effect relationship between increased oxidative stress, RAAS activity and increased blood pressure has remained unclear.

Therefore, the aim of this study was to examine and correlate the levels of MG, mediators of the renin angiotensin system and blood pressure in high fructose diet fed SD rats.

## Materials and Methods

### Animals

Male Sprague-Dawley rats from Charles River Laboratories (Quebec, Canada) were used according to guidelines of the Canadian Council on Animal Care. All animal protocols were approved by the University of Saskatchewan’s Animal Research Ethics Board. 32 male 5 week old SD rats were randomly divided into the following treatment groups (*n* = 8 in each group): 1. Control (normal rat chow), 2. High fructose diet (60% of total calories), 3. High fructose diet+metformin (500 mg/kg/day in drinking water, MG scavenger [26], [27]), 4. Metformin (normal chow). The treatments were for 16 weeks.

At the end of the treatment period, the rats were anaesthetized with sodium pentobarbital (60 mg/kg body weight, i.p.). The trachea was cannulated to allow spontaneous respiration. The carotid artery was cannulated and connected to a pressure transducer and a Powerlab system (AD Instruments Inc., Colorado Springs, CO, USA) using LabChart 7 software. After 15 min stabilization the blood pressure was recorded for 30 min [28]. Blood was collected from the carotid artery and plasma was separated and stored at −80°C. The anesthetized rat was euthanized by cutting the thorax and the heart, and causing exsanguination, as per the guidelines of the Canadian Council on Animal Care. Organs and tissues cleaned in ice-cold phosphate buffer saline were immediately frozen in liquid nitrogen and stored at −80°C until processing.

### Cell Culture

Rat thoracic aortic smooth muscle cells (A-10 cells, CRL-1476, American Type Culture Collection, Manassas, VA, USA) were cultured in Dulbecco’s Modified Eagle’s Medium (DMEM) containing 10% fetal bovine serum (FBS), 1% penicillin-streptomycin at 37°C in a humidified atmosphere of 95% air and 5% CO_2_, as described in our previous study [7]. The cells were seeded in 100 mm dishes, with an equal amount of cells (10^6^/ml) in each dish, and cultured to confluence. Cells were starved in FBS- free DMEM medium for 24 h prior to exposure to different treatments alone or in combination: MG (30 µM based on preliminary results), and metformin (100 µM).

### Methylglyoxal Measurement

MG was measured by a specific and sensitive high-performance liquid chromatography (HPLC) method as described earlier [29]. MG was derivatized with *o*-phenylenediamine to form the quinoxaline product, 2-methylquinoxaline, which is very specific for MG. The 2-methylquinoxaline and the internal standard 5-methylquinoxaline were quantified on a Hitachi D-7000 HPLC system (Hitachi, Ltd., Mississauga, ON, Canada) *via* a Symmetry C18 column (3.9×150 mm, and 4 µm particle diameter; Waters Corp., Milford, MA, USA).

### Western Blotting

Total proteins from cells, isolated aorta and kidney, were isolated with RIPA (lysis) buffer using a polytron homogenizer. The supernatants (40 µg of protein each) were separated by using 6–10% SDS-PAGE gel and subjected to Western blot analysis as previously described [12] with overnight incubation with primary antibodies to AT_1_ receptor for angiotensin (AT_1_R), adrenergic α_1D_ receptor (α_1D_R), renin, Ang II, (all from Santa Cruz Biotechnology Inc., Santa Cruz, CA, USA), receptor for AGEs (RAGE), nuclear factor κB (NF-κB) (Abcam, Cambridge, MA, USA), and anti-β-actin (diluted 1∶1000) (Sigma-Aldrich Canada Ltd., Mississauga, ON, Canada). This was followed by incubation with horse radish peroxidase conjugated secondary antibodies (Life Science, Hercules, CA, USA) (diluted 1∶3000) for 2 h at room temperature. The proteins were then visualized with ECL chemiluminescence reagent (GE Healthcare Life Sciences, Pittsburgh, PA, USA) and exposed to X-ray film (GE Healthcare Life Sciences, Pittsburgh, PA, USA).

### Real Time Quantitative PCR (RT-PCR)

Total RNA from the kidney and the aorta was isolated using RNA isolation kit (Qiagen, Germantown, MD, USA). The pre-designed primers for Ang II, AT_1_R, α_1D_R, renin and RAGE were purchased from Qiagen, (Germantown, MD, USA). The real-time PCR was performed in an iCycler iQ apparatus (Life Science, Hercules, CA, USA) associated with the ICYCLER OPTICAL SYSTEM software (version 3.1) using SYBR Green PCR Master Mix (Bio-Rad Laboratories Ltd., Mississauga, ON, Canada).

### Measurement of Reduced Glutathione (GSH)

Reduced glutathione was measured by derivatization with 5,5′-dithio-bis(2-nitrobenzoic acid), and reverse-phase HPLC using ultraviolet detection [6].

### Statistical Analysis

Data obtained from separate experiments are expressed as mean ± SEM. Statistical analysis was performed using ANOVA with post hoc Bonferroni’s test. A *P* value of less than 0.05 was considered to be statistically significant.

## Results

### Chronic Fructose Treatment Increases Blood Pressure, Aortic and Renal Methylglyoxal Levels

**Figure 1:**
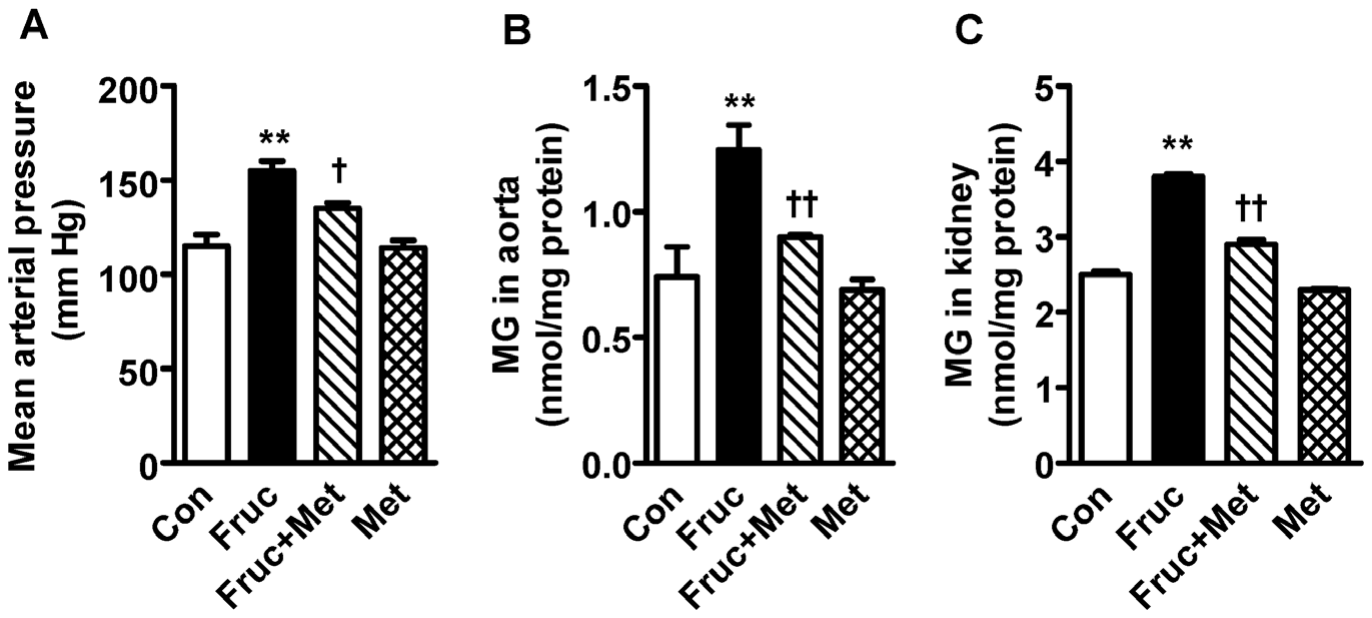
Mean arterial blood pressure and methylglyoxal (MG) levels in the aorta and kidney of high fructose diet treated Sprague-Dawley rats. Groups (*n = *8 each) of 5 week old male Sprague-Dawley rats were treated for 16 weeks with a high fructose diet (Fruc, 60% of total calories). Control rats received normal chow. Metformin (Met, 500 mg/kg/day in drinking water) was used as a MG scavenger. Mean arterial pressure was measured with an intra-carotid artery catheter in anesthetized rats. MG levels were determined with HPLC. **P<0.01 *vs*. respective control (Con). ^†^ *P*<0.05, ^††^ *P*<0.01 *vs*. respective Fruc group.

Treatment of SD rats with a high fructose diet for 16 weeks caused a significant increase in the blood pressure, which was attenuated by the MG scavenger metformin (Fig. 1A). Metformin alone did not affect the blood pressure. The high fructose diet also caused a significant elevation of aortic and renal MG levels, which was attenuated by co-treatment with metformin (Fig. 1B, C).

### Chronic Treatment with Fructose Increases Aortic α_1D_ Receptor, AT_1_ Receptor and Angiotensin II Expression

**Figure 2:**
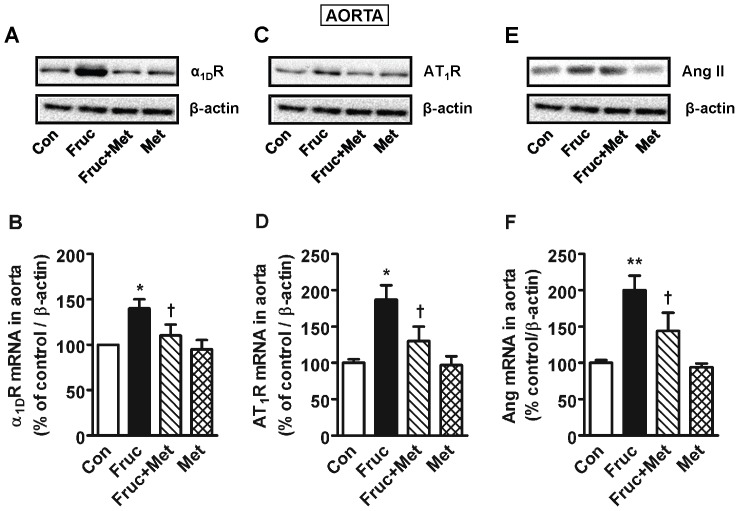
Adrenergic α1D receptor (α1DR), AT1 angiotensin receptor (AT1R) and angiotensin II (Ang II) expression in the aorta of high fructose diet treated Sprague-Dawley rats. Groups (*n = *8 each) of 5 week old male Sprague-Dawley rats were treated for 16 weeks with a high fructose diet (Fruc, 60% of total calories). Control rats received normal chow. Metformin (Met, 500 mg/kg/day in drinking water) was used as a MG scavenger. Protein expression was determined by Western blotting using appropriate primary antibodies, and mRNA with RT-PCR. *P<0.05, **P<0.01 *vs*. respective control (Con). ^†^ *P*<0.05 *vs*. respective Fruc group.

Chronic treatment of SD rats with fructose for 16 weeks significantly elevated aortic adrenergic α_1D_ receptor, angiotensin AT_1_ receptor and Ang II protein and mRNA, which were attenuated by co-treatment with metformin (Fig. 2). Metformin alone was without any effect.

### Chronic treatment of SD Rats with Fructose Increases Renal AT_1_ Receptor, Renin and Angiotensin II Expression

**Figure 3:**
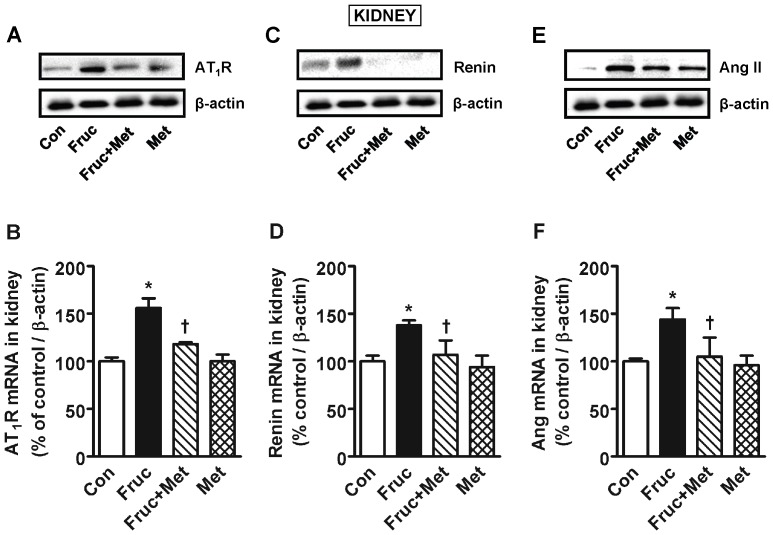
AT1 angiotensin receptor (AT1R), renin and angiotensin II (Ang II) expression in the kidney of high fructose diet treated Sprague-Dawley rats. Groups (*n = *8 each) of 5 week old male Sprague-Dawley rats were treated for 16 weeks with a high fructose diet (Fruc, 60% of total calories). Control rats received normal chow. Metformin (Met, 500 mg/kg/day in drinking water) was used as a MG scavenger. Protein expression was determined by Western blotting using appropriate primary antibodies, and mRNA with RT-PCR. *P<0.05 *vs*. respective control (Con). ^†^ *P*<0.05 *vs*. respective Fruc group.

Chronic treatment of SD rats with fructose for 16 weeks significantly increased renal AT_1_ receptor, renin and Ang II protein and mRNA, which were attenuated by co-treatment with metformin (Fig. 3). Metformin alone was without any effect.

### Chronic Treatment with Fructose Increases Phosphorylated Extracellular Signal Related Kinases 1/2 (p-Erk 1/2), and NFATc Expression

**Figure 4:**
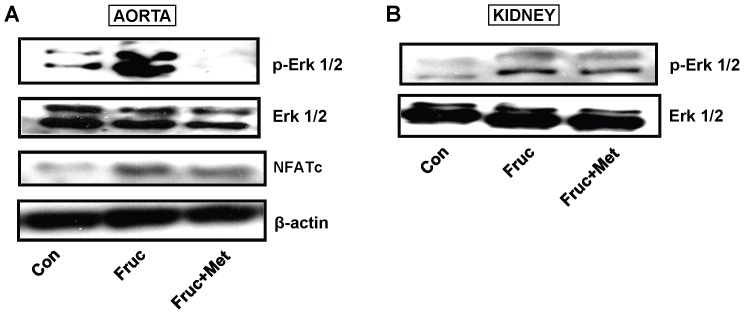
Phosphorylated extracellular signal-related kinases 1/2 (p-Erk 1/2), Erk 1/2 and NFATc expression in the aorta and kidney of high fructose diet treated Sprague-Dawley rats. Groups (*n = *8 each) of 5 week old male Sprague-Dawley rats were treated for 16 weeks with a high fructose diet (Fruc, 60% of total calories). Control rats received normal chow. Metformin (Met, 500 mg/kg/day in drinking water) was used as a MG scavenger. Protein expression was determined by Western blotting using appropriate primary antibodies.

Chronic treatment of SD rats with fructose for 16 weeks increased aortic and renal protein expression of phosphorylated Erk 1/2 (p-Erk 1/2 ) and NFATc in the aorta, which was attenuated by metformin (Fig. 4A, B).

### Chronic Treatment with Fructose Increases NF-κB and RAGE Protein Expression

**Figure 5:**
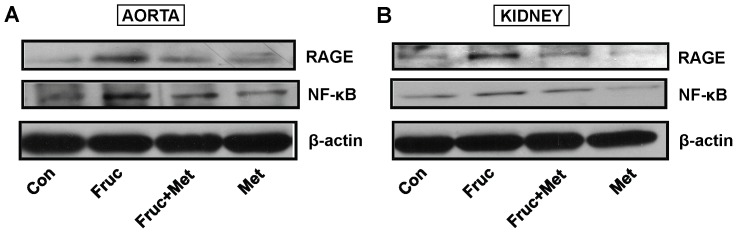
Nuclear factor κB (NF-κB) and receptor for advanced glycation endproducts (RAGE) expression in the aorta and kidney of high fructose diet treated Sprague-Dawley rats. Groups (*n = *8 each) of 5 week old male Sprague-Dawley rats were treated for 16 weeks with a high fructose diet (Fruc, 60% of total calories). Control rats received normal chow. Metformin (Met, 500 mg/kg/day in drinking water) was used as a MG scavenger. Protein expression was determined by Western blotting using appropriate primary antibodies.

Chronic treatment of SD rats with fructose for 16 weeks significantly increased aortic and renal NF-κB and RAGE protein expression, which was attenuated by co-treatment with metformin (Fig. 5A, B).

### Chronic Treatment with Fructose Decreases Levels of Reduced Glutathione in the Aorta and Kidney

**Figure 6:**
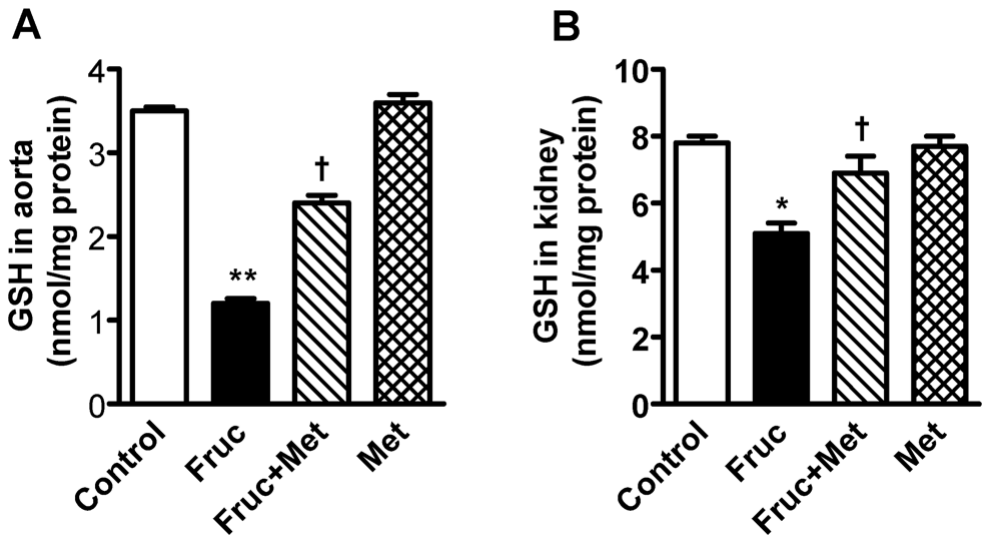
Reduced glutathione (GSH) levels in the aorta and kidney of high fructose diet treated Sprague-Dawley rats. Groups (*n = *8 each) of 5 week old male Sprague-Dawley rats were treated for 16 weeks with a high fructose diet (Fruc, 60% of total calories). Control rats received normal chow. Metformin (Met, 500 mg/kg/day in drinking water) was used as a MG scavenger. Glutathione levels were determined with HPLC. *P<0.05, **P<0.01 *vs*. respective control. ^†^ *P*<0.05, *vs*. respective Fruc group.

Chronic treatment of SD rats with fructose for 16 weeks significantly decreased aortic and renal levels of reduced glutathione, which were attenuated by co-treatment with metformin (Fig. 6A, B).

### Methylglyoxal Increases α_1D_ Receptor, AT_1_ Receptor and Angiotensin II Expression in Cultured Vascular Smooth Muscle Cells

**Figure 7:**
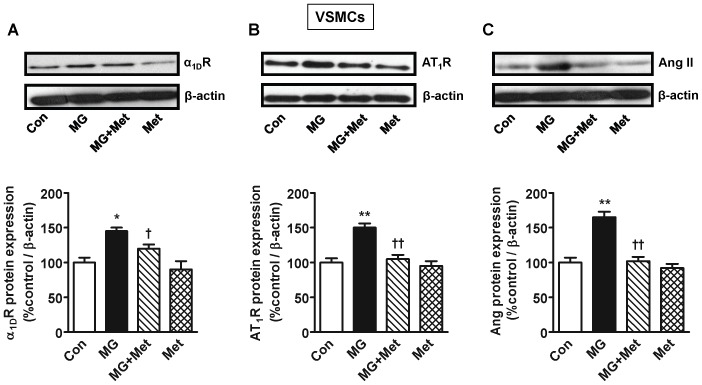
Angiotensin II (Ang II), AT1 receptor (AT1R) and α1D receptor (α1DR) expression in cultured vascular smooth muscle cells (VSMCs) treated with methylglyoxal. Rat thoracic aorta smooth muscle cells (A10 cell line) were cultured and incubated with MG (30 µM) for 24 h. Metformin (100 µM) was used as a MG scavenger. Protein expression was determined by Western blotting using appropriate primary antibodies. *n* = 4 for each group. *P<0.05, **P<0.01 *vs*. respective control (Con). ^†^ *P*<0.05, ^††^ *P*<0.01 *vs*. respective MG group.

Treatment of rat thoracic aortic cultured vascular smooth muscle cells with MG (30 µM) for 24 h significantly elevated adrenergic α_1D_ receptor, angiotensin AT_1_ receptor and Ang II protein, which were attenuated by co-treatment with metformin (Fig. 7). Metformin alone was without any effect.

### Metformin has MG Scavenging Ability

As shown in table 1, 100 µM metformin significantly reduced the detectable free MG in solution after 3 h incubation, and about 30% of MG was detectable after 24 h incubation. A higher concentration of metformin (1 mM) was more effective in scavenging MG and it significantly reduced detectable free MG after 1 h of incubation and only about 10% of MG was detectable after 24 h.

**Table 1:** *In vitro* assay to determine the methylglyoxal (MG) scavenging ability of metformin.

Incubationtime	MG (30 µM)	MG (30 µM)+metformin(100 µM)	MG (30 µM)+metformin(1 mM)	Metformin(100 µM)	Metformin(1 mM)
15 min	24.89±0.15	21.69±0.17	18.79±0.21	0	0
30 min	25.28±0.21	19.29±0.06	15.28±0.32	0	0
60 min	26.82±0.15	17.87±0.31	10.38±0.51*	0	0
3 h	25.77±0.14	12.52±0.08*	7.36±0.27**	0	0
24 h	25.23±0.31	9.43±0.41**	3.39±0.32***	0	0

MG was incubated with metformin at different concentrations at 37°C for different times. The solution was analyzed for free MG by HPLC after the given incubation period. The values (µM) are expressed as mean ± SEM (*n* = 6 in each).

## Discussion

Here we show that 12 week old male SD rats treated for 16 weeks with a high fructose diet, a precursor of MG, develop a significant increase in blood pressure. The kidney and aorta from fructose treated rats had significantly increased MG levels, and protein and mRNA for renin, Ang II, AT_1_ and adrenergic α_1D_ receptors, which were attenuated by the MG scavenger metformin. MG treated cultured vascular smooth muscle cells had increased expression of Ang II, AT_1_ and α_1D_ receptors, which was attenuated by metformin.

Although high glucose [30] and high fructose diets [5], [31] have been shown to significantly increase the blood pressure in animals and humans, the molecular mechanisms are not very clear. A high glucose diet-induced increase in blood pressure has been attributed to activation of protein kinase C, increased oxidative stress and reduced bioavailability of nitric oxide [32]. Fructose is metabolized differently than glucose with up to 75% of orally absorbed fructose being metabolized by the liver [33]. Moreover, the key enzyme of fructose metabolism, fructokinase is not feedback regulated by fructose metabolites [33]. Thus, all of the orally absorbed fructose is phosphorylated by fructokinase [33], [34]. Unregulated metabolism of fructose causing ATP depletion, oxidative stress and decrease in nitric oxide production has been proposed as one of the mechanisms of fructose-induced increase in blood pressure [35], [36]. Both glucose and fructose are precursors of MG formation [7] and MG is also a well-established trigger for increased oxidative stress through multiple pathways [25]. MG is also a major precursor of the formation of AGEs [13], [14].

The aorta and the kidney of fructose treated rats had significantly elevated MG levels (Fig. 1) which implicates MG as a factor in hypertension development. The kidney had increased protein expression of renin, which in turn would produce more Ang II and its multiple effects [24]. The increased aortic MG, as observed in the present study, most likely increased aortic AT_1_ receptor, α_1D_ receptor and Ang II. This effect of MG was confirmed by increased expression of AT_1_ receptor, α_1D_ receptor and Ang II in MG treated cultured vascular smooth muscle cells (Fig. 7). Both AT_1_ and α_1D_ receptor activation can increase p-Erk 1/2, and NFATc, as seen here, which can contribute to increased vascular contractility, inflammation and hypertension development [24], [37]. The increased Ang II may be responsible for the increased α_1D_ receptor expression [22], [23]. The increased Ang II, AT_1_ and α_1D_ receptors in the aorta signify increased vascular tone. We were not able to harvest enough tissue from the mesenteric artery for western blot and PCR analysis. GSH levels were reduced in the aorta and the kidney of fructose treated rats (Fig. 6). GSH plays a central role in the degradation of MG by binding MG and making it available to the glyoxalase enzymes [14]. A reduction in GSH would decrease MG degradation, increase its levels, and set up a vicious cycle.

We addressed the possibility that MG was increasing oxidative stress, which has been implicated in the pathogenesis of hypertension [18]. The effect of MG on RAGE has not been reported before. RAGE is currently under intense investigation as a target to prevent diabetic complications. The activation of RAGE by AGEs has been reported to increase two key transcription factors, NF-κB and early growth response-1 (Egr-1), and cause oxidative stress [38], [39]. We observed increased RAGE and NF-κB in the aorta and the kidney of fructose treated rats. These results suggest that fructose treatment increases MG levels, which in turn can activate RAGE, which then increases NF-κB and oxidative stress. The increased oxidative stress increases expression of Ang II, AT_1_ and α_1D_ receptors. The increased Ang II in turn can increase NF-κB and oxidative stress [24] and set up a vicious cycle. Our results suggest a possible sequence of molecular events resulting from elevated endogenous MG levels which have been reported in high carbohydrate diet fed animals and in diabetic patients [5], [6], [10], [11].

Metformin is used clinically as an oral anti-diabetic drug in patients with type 2 diabetes. Metformin has multiple effects *in vivo*. For example, it inhibits mitochondrial respiration and gluconeogenesis in the liver, activates AMP-activated protein kinase, increases insulin sensitivity, antagonizes the action of glucagon and increases fatty acid oxidation [40]. These *in vivo* antidiabetic actions of metformin can make interpretation of results difficult, making it a less than ideal experimental MG scavenger. Even though the MG scavenging ability of metformin has been studied in specific experiments and reported before [26], [27], we tested the MG scavenging ability of metformin in cultured vascular smooth muscle cells treated with MG, where multiple *in vivo*, especially hepatic, actions of metformin do not come into play. We also performed an *in vitro* MG scavenging assay for metformin. In cultured vascular smooth muscle cells metformin attenuated the increased expression of α_1D_ and AT1 receptors, and Ang II (Fig. 7). These results support a direct effect of MG on these RAAS mediators and the MG scavenging action of metformin. As shown in table 1 metformin displayed significant MG scavenging ability when the two were mixed in solution.

## Conclusions

In conclusion, high fructose diet fed rats had significantly elevated blood pressure, MG levels in the aorta, and the kidney, and increased expression of Ang II, AT_1_ receptor, α_1D_ receptor, renin, RAGE and NF-κB and decreased levels of reduced glutathione in the aorta and/or the kidney. The MG scavenger metformin attenuated these effects. Our results show a strong association between elevated levels of methylglyoxal, RAGE, NF-κB, mediators of the renin angiotensin system and blood pressure in high fructose diet fed rats. Therefore, it is possible that MG could be a mediator of high fructose diet-induced hypertension, probably acting through RAGE and NF-κB, and upregulating the renin angiotensin system.
